# Effect of aerobic exercise training on the fat fraction of the liver in persons with chronic hepatitis B and hepatic steatosis: Trial protocol for a randomized controlled intervention trial— The FitLiver study

**DOI:** 10.1186/s13063-023-07385-y

**Published:** 2023-06-13

**Authors:** Sofie Jespersen, Peter Plomgaard, Sten Madsbad, Adam Espe Hansen, Thomas Bandholm, Bente Klarlund Pedersen, Christian Ritz, Nina Weis, Rikke Krogh-Madsen

**Affiliations:** 1 The Centre for Physical Activity Research, Copenhagen University Hospital, Rigshospitalet, Copenhagen, Denmark; 2 The Department of Infectious Diseases, Copenhagen University Hospital, Hvidovre, Denmark; 3 The Department of Clinical Biochemistry, Copenhagen University Hospital, Rigshospitalet, Copenhagen, Denmark; 4 The Department of Clinical Medicine, Faculty of Health and Medical Sciences, University of Copenhagen, Copenhagen, Denmark; 5 The Department of Endocrinology, Copenhagen University Hospital, Hvidovre, Denmark; 6 The Department of Radiology, Copenhagen University Hospital, Rigshospitalet, Copenhagen, Denmark; 7 The Department of Physical and Occupational Therapy, Copenhagen University Hospital, Hvidovre, Denmark; 8 The Department of Clinical Research, Copenhagen University Hospital, Hvidovre, Denmark; 9 The National Institute of Public Health, University of Southern Denmark, Copenhagen, Denmark

**Keywords:** Hepatitis B, Fatty liver, Magnetic resonance imaging, Exercise, High-intensity interval training, Randomized controlled trial

## Abstract

**Background:**

The global prevalence of chronic hepatitis B is more than 300 million people, and in Denmark, 17,000 people are estimated to have chronic hepatitis B. Untreated, chronic hepatitis B can lead to the development of liver cirrhosis and liver cancer. There is no curable therapy. In persons with obesity and chronic hepatitis B infection, the development of hepatic steatosis imposes a double burden on the liver, leading to an increased risk of cirrhosis and liver cancer. In patients without chronic hepatitis B, exercise interventions have shown beneficial effects on hepatic steatosis through improvements in fat fraction of the liver, insulin resistance, fatty acid metabolism, and glucose metabolism, as well as activation of liver-induced regulatory protein secretion (hepatokines) after the exercise intervention.

**Objective:**

To investigate in persons with chronic hepatitis B and hepatic steatosis:

- Primary: Whether exercise will decrease the fat fraction of the liver.
- Secondary: If exercise will affect hepatokine secretion and if it will improve lipid- and glucose metabolism, liver status, markers of inflammation, body composition, and blood pressure.

**Methods:**

A randomized, controlled, clinical intervention trial consisting of 12 weeks of aerobic exercise training or no intervention. Thirty persons with chronic hepatitis B and hepatic steatosis will be randomized 1:1. Before and after the intervention, participants will undergo an MRI scan of the liver, blood sampling, oral glucose tolerance test, fibroscan, VO2_max_ test, DXA scan, blood pressure measurements, and optional liver biopsy. Lastly, a hormone infusion test with somatostatin and glucagon to increase the glucagon/insulin ratio for stimulating secretion of circulating hepatokines will be performed. The training program includes three weekly training sessions of 40 min/session over 12 weeks.

**Discussion:**

This trial, investigating high-intensity interval training in persons with chronic hepatitis B and hepatic steatosis, is the first exercise intervention trial performed on this group of patients. If exercise reduces hepatic steatosis and induces other beneficial effects of clinical markers in this group of patients, there might be an indication to recommend exercise as part of treatment. Furthermore, the investigation of the effect of exercise on hepatokine secretion will provide more knowledge on the effects of exercise on the liver.

**Trial registration:**

Danish Capital Regions committee on health research ethics reference: H-21034236 (version 1.4 date: 19–07-2022) and ClinicalTrials.gov: NCT05265026.

## Administrative information

**Table:** 

Title {1}	Effect of aerobic exercise training on the fat fraction of the liver in persons with chronic hepatitis B and hepatic steatosis: Trial protocol for a randomized controlled intervention trial - The FitLiver study
Trial registration {2a and 2b}	ClinicalTrials.gov: NCT05265026
Protocol version {3}	Version 1.4 was approved on July 19th, 2022
Funding {4}	The Beckett Foundation, The Holm Memory Foundation, The Department of Infectious Diseases, Copenhagen University Hospital, Hvidovre, Denmark The Centre for Physical Activity Research which is supported by TrygFonden (grants ID 101390, ID 20045, and ID 125132)
Author details {5a}	Sofie Jespersen*^1,2^, Peter Plomgaard^3,4^, Sten Madsbad^4,5^, Adam Espe Hansen^4,6^, Thomas Bandholm^4,7,8^, Bente Klarlund Pedersen^1,4^, Christian Ritz^9^, Nina Weis^2,4^, Rikke Krogh-Madsen^1,2,4^ Affiliations 1: The Centre for Physical Activity Research, Copenhagen University Hospital, Rigshospitalet, Copenhagen, Denmark 2: The Department of Infectious Diseases, Copenhagen University Hospital, Hvidovre, Denmark 3: The Department of Clinical Biochemistry, Copenhagen University Hospital, Rigshospitalet, Copenhagen, Denmark 4: The Department of Clinical Medicine, Faculty of Health and Medical Sciences, University of Copenhagen, Copenhagen, Denmark 5: The Department of Endocrinology, Copenhagen University Hospital, Hvidovre, Denmark 6: The Department of Radiology, Copenhagen University Hospital, Rigshospitalet, Copenhagen, Denmark 7: The Department of Physical and Occupational Therapy, Copenhagen University Hospital, Hvidovre, Denmark 8: The Department of Clinical Research, Copenhagen University Hospital, Hvidovre, Denmark 9: The National Institute of Public Health, University of Southern Denmark, Copenhagen, Denmark *corresponding author
Name and contact information for the trial sponsor {5b}	Investigator-initiated trial: Sofie Dikeledi Kold Jespersen (principal investigator), email: sofie.dikeledi.kold.jespersen@regionh.dk
Role of sponsor {5c}	This study is initiated by the investigators and the sponsor is thereby the institution where the primary investigator is appointed: Centre of Physical Activity Research, Copenhagen University Hospital, Rigshospitalet, Copenhagen, Denmark. The sponsor holds the indemnity insurance and legal liability. The funders played no role in the study design or the collection, analysis, and interpretation of data and writing of the manuscript

## Introduction

### Background and rationale {6a}

Hepatitis B virus (HBV) causes chronic hepatitis B (CHB), which is defined as hepatitis B surface antigen (HBsAg) in the blood > 6 months. The estimated global prevalence of CHB is estimated to be 316 million individuals [1], and CHB is the most common type of hepatitis in the world. Untreated, CHB can lead to the development of liver cirrhosis and cancer, known as hepatocellular carcinoma (HCC). There is at present no curable therapy, but medical treatment can reduce the amount of virus in the blood, leading to reduced CHB-induced morbidity and mortality.

#### Comorbidity in persons with CHB

Persons with CHB have a higher body mass index (BMI), consume more alcohol, and have poorer physical fitness compared to uninfected [2], and approximately 33% are overweight and 50% physically inactive [3]. In general, especially central obesity is associated with the metabolic syndrome (i.e. central obesity, hypertension, glucose abnormality, and dyslipidemia) and thereby increased risk of development metabolic diseases such as type 2 diabetes (T2DM) and cardiovascular disease (CVD) [4]. Metabolic dysfunction is associated with non-alcoholic fatty liver disease (NAFLD) [5, 6]. NAFLD is projected to have a prevalence of > 30% in Asia and the USA populations [7, 8]. NAFLD is defined by the presence of steatosis > 5% in the hepatocytes [9] but excludes the diagnosis of hepatitis B. Hence, this trial will refer to the term hepatic steatosis when describing fatty liver disease in persons with CHB. The prevalence of concomitant hepatic steatosis and CHB vary dependent on degree of obesity, hypertriglyceridemia, and T2DM [10, 11]. In a meta-analysis including 21 studies comprising 4100 persons with CHB, the prevalence of biopsy-proven hepatic steatosis was 29.6% [12]. Other studies have indicated a prevalence of 14–30% [13], and a recent meta-analysis including 98 studies using a variety of ultrasound, liver biopsies, controlled attenuated parameter and magnetic resonance spectroscopy, to assess hepatic steatosis in persons with CHB, a prevalence of 35% was reported [14]. In persons with obesity and CHB, the development of hepatic steatosis imposes a double burden on the liver. Having CHB and hepatic steatosis have been shown to increase the risk of cirrhosis and HCC [15, 16].

#### Systemic inflammation in persons with CHB and comorbidity

Obesity is associated with chronic systemic low-grade inflammation, which is the key point in the initiation and progression of obesity-related NAFLD [17–19]. Chronic systemic low-grade inflammation is characterized by a two- to fourfold elevation in circulating levels of inflammatory cytokines and acute-phase reactants [20]. One of the main mechanisms is thought to be increased inflammation of the adipose tissue [21]. Circulating levels of tumor necrosis factor (TNF)-alpha and interleukin (IL)-6 are increased in chronic HBV infection and are positively associated with disease progression (hepatitis B progression to cirrhosis or HCC) [22, 23]. Whether chronic inflammation is due to HBV infection, comorbidities like obesity and T2DM, or both is unclear [24].

#### Hepatokines in persons with CHB

Another unclear mechanism is the signalling pathways of hepatokines. Hepatokines are proteins secreted by hepatocytes, and several hepatokines have been associated with metabolic dysfunctions [25, 26]. Hepatokines are suggested to change during HBV infection. Increased plasma levels of the hepatokine Fibroblast Growth Factor 21 (FGF-21) are associated with hepatic steatosis [27], but low levels of FGF-21 are associated with advanced fibrosis/cirrhosis in patients with CHB. FGF-21 is regulated by glucagon as well as macronutrients and is increased after exercise [28]. Another hepatokine, follistatin, plays a critical role in hepatocyte regeneration during the repair of liver tissue [29] and is also a part of the process of muscle hypertrophy [30]. Follistatin has an inverse correlation with HBV DNA levels [31]. The hepatokine angiopoeitin like protein 4 (ANGPTL4) and growth differentiation factor-15 (GDF-15) is secreted from the liver during exercise [32, 33] and ANGPTL4 is involved in regulating plasma triglyceride levels [34]. In patients with HBV-associated HCC GDF-15 is found to be increased and have an association with alpha-fetoprotein [35]. No studies have yet explored ANGPTL4’s relation to CHB. Studies investigating the secretion of the hepatokines are warranted to understand how the pathways are regulated and which target areas future medical treatment should focus on.

#### Effects of exercise

Exercise interventions of 6 weeks or more show beneficial effects on NAFLD patients without CHB through improvements in the fat fraction of the liver [36, 37], liver and peripheral insulin resistance, liver fatty acid metabolism and activation of anti-inflammatory cascades after the physical exercise intervention [38]. The anti-inflammatory properties of exercise are multifactorial. At least due to both a reduction in visceral adipose tissue and a more direct induction of anti-inflammatory, immune cells.

IL-6 is a cytokine implicated in the regulation of energy metabolism [39, 40]. IL-6 can be persistently elevated in individuals with obesity [41] and acutely following infection [42] and exercise [43, 44]. In exercise settings, IL-6 is not preceded by an increase in plasma TNF-alpha as seen during infections. During exercise, IL-6 is produced by contracting muscle fibers and released as a myokine into the bloodstream [44, 45]. It has been suggested that IL-6 could suppress HBV replication and inhibit HBV entry [45, 46]. The possible beneficial effect of the exercise-induced increase in circulating IL-6 in chronic HBV infection is unknown.

It is important to clarify the role of aerobic exercise training in preventing developing comorbidity and treating lifestyle-induced conditions like obesity, glucose tolerance, and fatty liver disease. Furthermore, it is necessary to understand the metabolic mechanism of activating inflammatory cascades and signalling through cytokines and hepatokines to improve targeting treatment of the double burden of chronically diseased persons. To our knowledge, no randomized exercise intervention trial has ever been made in persons with CHB. We wish to conduct a randomized controlled intervention trial to investigate the effects of aerobic exercise training on persons with CHB and hepatic steatosis to investigate the changes in the fat fraction of the liver and how aerobic exercise training alternates conditions related to the metabolic syndrome and cytokine and hepatokine secretion.

### Objectives {7}

The main objective is to investigate if regular aerobic exercise training can decrease the amount of fat in the liver in persons with CHB and hepatic steatosis.

A secondary objective is to investigate the effects of aerobic exercise training on hepatokine secretion in persons with CHB and hepatic steatosis. We also aim to investigate if regular aerobic exercise training will reduce obesity and improve lipid and glucose metabolism, liver status—such as elevated ALT and INR, markers of inflammation, body composition, and blood pressure in persons with CHB and hepatic steatosis.

#### Hypothesis

##### Primary hypothesis

Regular aerobic exercise training decreases the fat fraction of the liver.

##### Secondary hypothesis

Exercise-induced hepatokine release, glucose, and lipid metabolism are compromised in persons with CHB and hepatic steatosis and benefit from aerobic exercise training.

### Trial design {8}

**Fig. 1:**
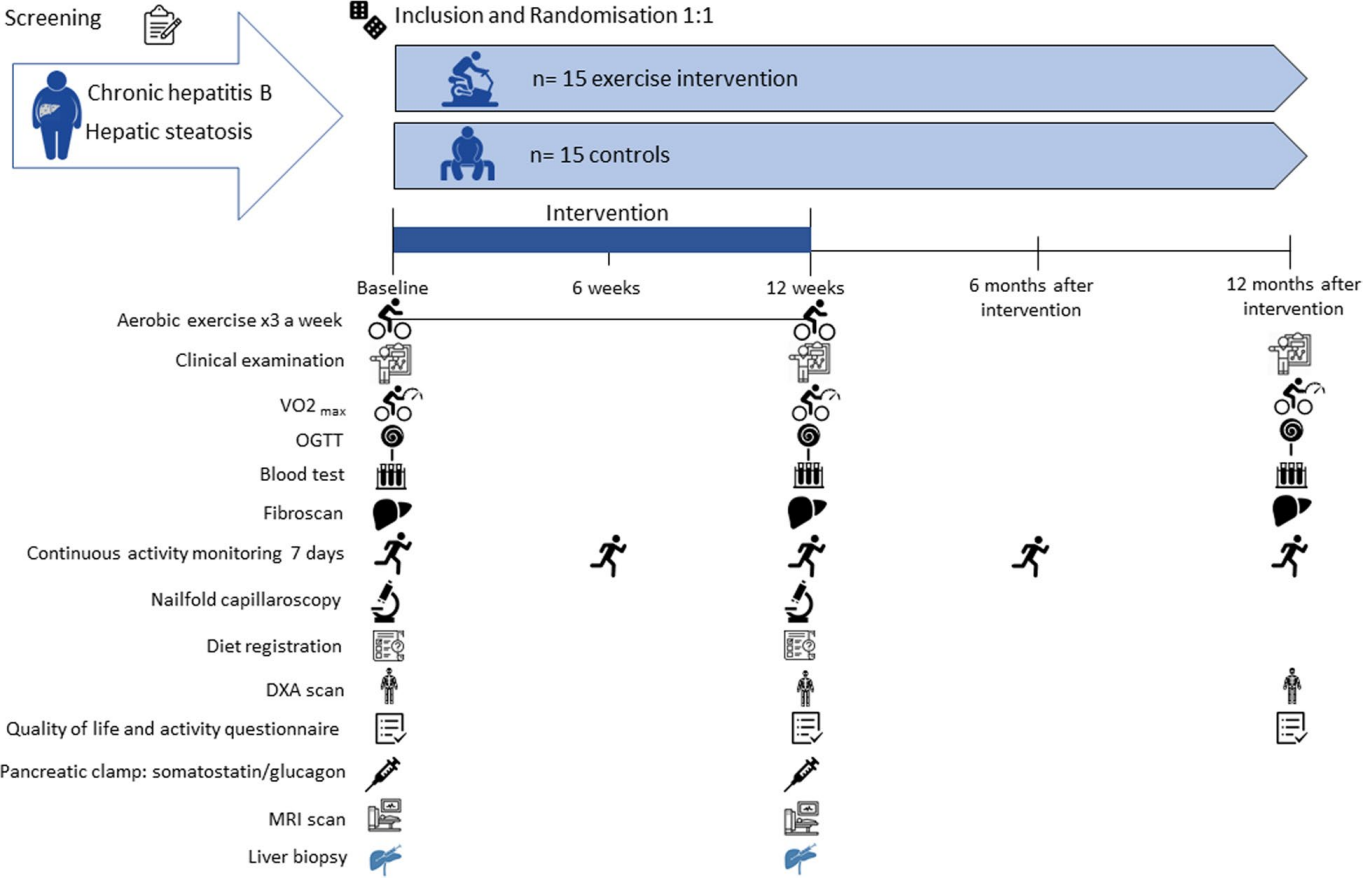
Study design

This is an investigator-initiated trial that was registered at www.ClinicalTrials.gov before inclusion of the first participant. The trial is designed as a block-randomized, controlled, unblinded, clinical, superiority, exploratory, intervention trial. Thirty persons with CHB and hepatic steatosis are randomized 1:1 to either aerobic exercise training (intervention group, *n* = 15) or no intervention (“do-as-usual” control group, *n* = 15). See Fig. 1 for the study design. The trial will be reported using the CONSORT extension for non-pharmacological treatment interventions [47] and key elements from the REPORT Trial guide [48].

## Methods: participants, intervention, and outcomes

### Study setting {9}

The trial visits will take place at the Centre for Physical Activity Research (CFAS), Copenhagen University Hospital, Rigshospitalet with magnetic resonance imaging (MRI) examinations at the Department of Radiology at Copenhagen University Hospital, Rigshospitalet. The liver biopsy (not mandatory) and the fibroscans will be performed at the Department of Infectious Diseases, Copenhagen University Hospital, Hvidovre. The aerobic exercise training sessions will be carried out at CFAS, or at the Department of Physio- and Occupational Therapy at Copenhagen University Hospital, Hvidovre.

### Eligibility criteria {10}

**Fig. 2:**
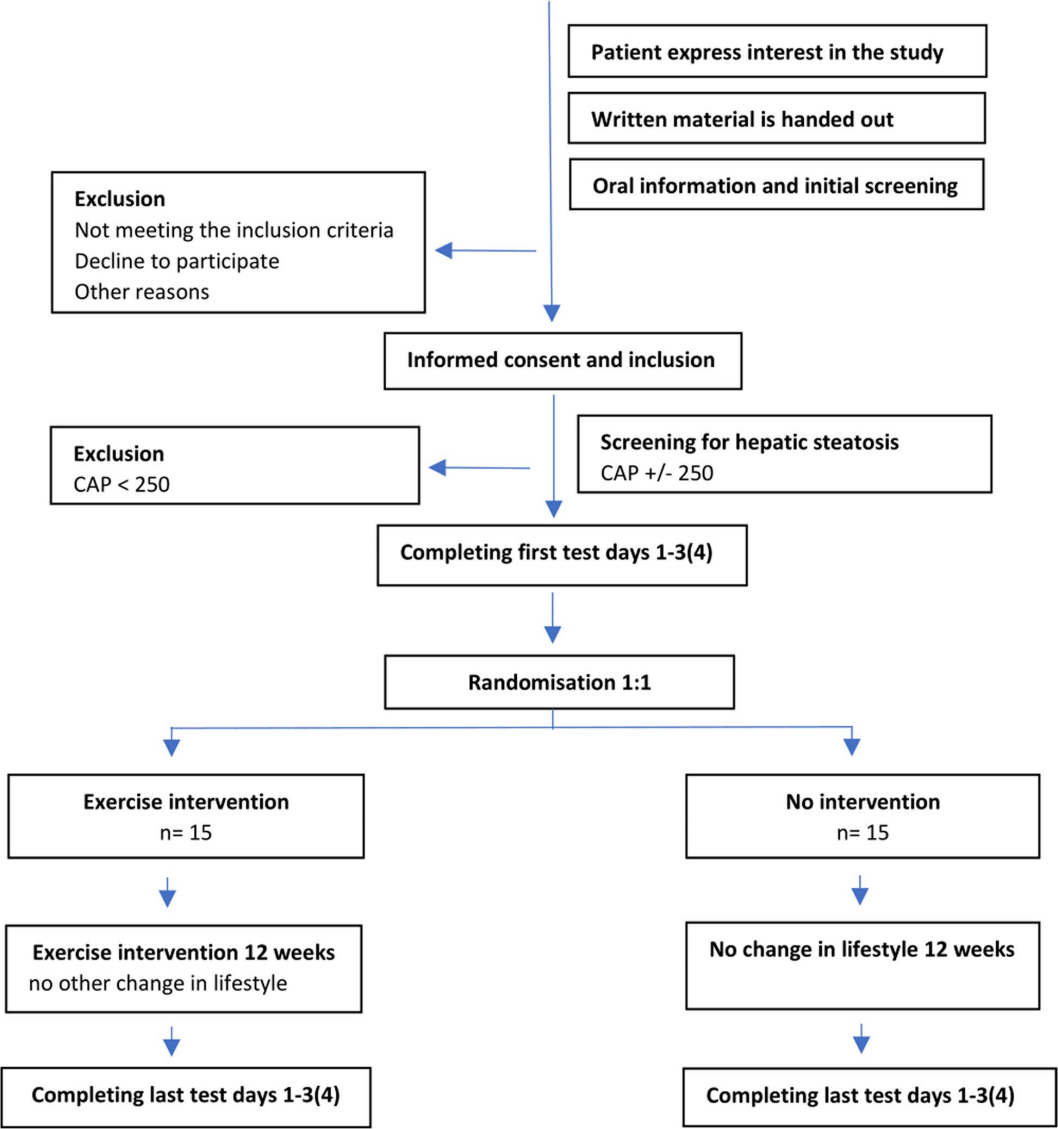
Flowchart of participants

Trial participants will be recruited at three departments in Copenhagen University Hospital Denmark at a regular outpatient visit: the Department of Infectious Diseases, Copenhagen University Hospital, Hvidovre, the Department of Infectious Diseases, Copenhagen University Hospital, Rigshospitalet, and the Department of Internal Medicine, Copenhagen University Hospital, Herlev; all departments are part of hospitals in the Copenhagen metropolitan area in Denmark. Recruitment posters will be posted online, and participants will be able to contact the principal investigator if interested. See Fig. 2 for flowchart.

#### Criteria of inclusion

- CHB defined by HBsAg positive > 6 months
- Positive Hepatitis B virus-DNA
- Age ≥ 30 years (no upper age limit)
- Hepatic steatosis diagnosed by controlled attenuated parameter (CAP) > 250 assessed by transient elastography or by ultrasound-defined hepatic steatosis

#### Criteria of exclusion

- Human immunodeficiency virus (HIV), hepatitis C virus (HCV), or hepatitis D virus (HDV) co-infection
- Primary biliary cholangitis
- Wilson’s disease
- Autoimmune hepatitis
- Hepatocellular carcinoma
- Antiviral medication
- Steatogenic medication (systemic corticosteroids, amiodarone, tamoxifen, valproic acid, and methotrexate)
- Contraindications for MRI scan
- Average daily alcohol intake > 30 g for men and > 20 g for women
- Coronary artery disease contraindicating HIIT
- Unable to understand and read written information for participant’s written consent
- Pregnancy

Since the liver biopsy is optional, participants with contraindications for a liver biopsy, can be included in the study without having a liver biopsy performed.

### Who will take informed consent? {26a}

The primary investigator or a project nurse will take the informed consent.

### Additional consent provisions for collection and use of participant data and biological specimens {26b}

On the consent form, it is stated in a separate paragraph that participants give consent to the collection of blood plasma to be stored in a research biobank. The biobank is under the legal supervision of the Danish Data Protection Regulation. Any future research using the plasma will be approved by the Danish Capital Regions Committee on health research ethics and, if required by the committee, the participants will give their consent again for a specific project.

## Interventions

### Explanation for the choice of comparators {6b}

We choose to do a randomized controlled clinical trial involving persons with CHB and hepatic steatosis where the comparators were: The persons in the exercise intervention group and the “do-as-usual” controls. Since no previous clinical trials have investigated the effects of aerobic exercise training in persons with CHB, we found it meaningful to use non-exercising comparators to get an estimation of the pure effects of exercise.

### Intervention description {11a}

The intervention description below follows the Consensus on Exercise Reporting Template (CERT) [49].

#### The exercise and equipment

The exercise intervention group will undergo a supervised exercise program. The exercise program includes three weekly supervised aerobic exercise training sessions of 40 min per session over 12 weeks. The program consists of high-intensity interval training on ergometer bicycles from TechnoGym (Pedan A/S, Copenhagen, Denmark) and Monark (Monark Exercise AB, Denmark) which allow monitoring and adjustment of watt. The training consists of a warm-up of 10 min, where the first 5 min are at 60–69% maximum heart rate (HR_max_) then additional 5 min at 70–79% HR_max_, followed by 25 min of high-intensity interval training, and finally a 5 min cool-down. The high-intensity interval training will be done by 4 × 4 using intervals lasting 4 min performed 4 times with 3 min restitution in between. Previous studies in CFAS have had significant results following this training program [50, 51]. The maximal oxygen consumption rate (VO2_max_) will be performed pre- and post-exercise/no-exercise intervention and will determine the relative workload accordingly. To ensure proper intensity, all patients will be wearing a heart rate monitor (Polar heart rate monitors and physical fitness watches). Since there is a good correlation between VO2_max_ and HR_max_ [52], this is a practical method to monitor the internal training load. The maximal heart rate (HR_max_) will be measured as the peak heart rate during the final minute of the VO2_max_ test [53]. Heart rate monitoring is one of the most used methods to monitor internal training load [54]. The exercise program follows the current ACSM guidelines for exercise [55] and is chosen because the exercise of similar intensity and duration is known to result in IL-6 release [55, 56] and lead to changes in VAT [55, 57].

#### Qualifications of instructors

All training sessions will be supervised and administered by CFAS personnel (students of sports science, physiotherapy students, or medical students). All CFAS personnel have attended a training session, and at least 2 supervised sessions by experienced trainers, before supervising the sessions alone.

#### Individual and small group sessions

The training sessions will mostly be performed in an individual setting, but up to 4 participants can be trained at the same time, each participant with different startup times in order to be supervised by the CFAS personnel.

#### Supervision

The supervision of the aerobic exercise training will be done by the CFAS personnel, who will physically stand next to the participant throughout the exercise session, checking the heart rate of the participant and the watts on the ergometer bike. According to the participants’ wishes, the CFAS personnel will encourage and assist with adjustment of the bike and intensity of the exercise and observe the participant when exercising.

#### Adherence to intervention and fidelity

To evaluate the precise amount of exercise completed for every patient, each training session will be documented (attendance, total time of HR_max_ > 85%, total time of exercise, average wattage, and average heart rate). The Borg Rating of Perceived Exertion scale of 6–20 [58] is used to assess the intensity of the exercise training session perceived by the participant.

#### Motivation strategies

At each exercise training session, the participant is encouraged to perform as well as possible and is complemented on their achievements and progressions during the intervention period. If there is more than one cancellation of exercise training sessions, a motivational call will be made by the primary investigator to identify complications and challenges.

#### Exercise progression

As the intervals follow the HR_max_, the watt intensity will progress throughout the 12 weeks of training.

#### Additional components

There are no home program components or non-exercise components in this trial. To control factors that may influence the intervention’s effect, the participants are instructed not to change their lifestyle habits throughout the intervention.

#### Adverse events

These will be reported at each training session.

#### Setting

The exercise training sessions will be carried out at the facilities at CFAS and at the Department of Physical and Occupational Therapy, Copenhagen University Hospital, Hvidovre.

The participants in the control group are instructed to continue their life unchanged and will receive a standard of care in the healthcare system. Standard of care or usual care includes yearly or half-yearly visits to the Department of Infectious Diseases for control of their chronic viral disease with blood tests, fibroscan, and consultation with doctors and nurses. Also, any other treatment participants receive for any other chronic or acute disease at their general practitioner or outpatient clinic. No diet or exercise intervention is offered to the control group.

### Criteria for discontinuing or modifying allocated interventions {11b}

The participants will be randomly allocated to a group (control /exercise) after the initial baseline test is performed. Sufficient compliance to the exercise intervention is defined by the compliance criteria: Completing ≥ 70% of the 36 exercise training sessions with ≥ 50% of the assigned heart rate max (HR_max_). No participant will be allocated to a different arm after randomization. If a participant is given hepatic steatosis-inducing medicine or diagnosed with HIV/hepatitis C during the trial, they will be taken out of the trial.

### Strategies to improve adherence to interventions {11c}

Participants in the exercise group will be monitored doing the intervention by a trainer and encouraged to perform according to guidelines. If a participant does not comply with the prescribed exercise training in two connecting sessions, the primary investigator will be informed, and the participant will be contacted to identify the challenges and find a solution with the participant to improve adherence.

### Relevant concomitant care permitted or prohibited during the trial {11d}

All concomitant care is allowed during the trial.

### Provisions for post-trial care {30}

All patients will receive unchanged standard medical care by the Danish public health care system. If any clinically relevant findings occur during the trial the participant will be referred to the relevant department for clarification and treatment. All participants will be covered by The Danish Patient Insurance Association for any injury that may occur as a direct consequence of trial-related procedures.

### Outcomes {12}

#### Primary outcome assessed at week 13 after randomization

The primary outcome is the fat fraction of the liver (%). A fat fraction > 5% is considered hepatic steatosis; having hepatic steatosis is considered a risk factor for the development of hepatocellular carcinoma [9, 15]. Thus, a normalization of the fat fraction in the liver < 5% would be considered a risk reduction.

The primary outcome is assessed by MRI measurements of the proton density fat fraction [59, 60] (1.5 Tesla field strength GE Signa Artist-scanner. IDEAL-IQ sequence).

#### Secondary outcomes assessed at week 13 after randomization

- Hepatokine secretion during a somatostatin and glucagon hormone infusion (ANGPTL-4, follistatin, GDF15, FGF-21)
- Cytokine secretion during a hormone infusion (IL-6, IFN-γ, TNF-α, IL-1β, IL-4, IL-8, IL-10)
- Measurement of visceral fat (kg) based on Dixon MRI of the abdomen
- Body composition (fat and lean mass (kg)) assessed by dual-energy X-ray absorptiometry (DXA) scans
- Measurement of body weight (kg), height (cm), hip and waist circumference (cm) calculating body mass index (kg/m^2^) and waist to height ratio
- Blood pressure measurements (systolic (mmHg) and diastolic (mmHg))
- Physical fitness by VO2_max_ (mL/kg/min) performed on ergometer bikes
- Physical activity monitoring is assessed by how many hours a participant is sedentary or active (hours: minutes/week) measured by the use of accelerometers AX3 axivity
- OGTT measurements (glucose (mmol/L), insulin (pmol/L), c-peptide (pmol/L)): the amount of normal OGTT (%) and mathematical modelling of endocrine pancreas alpha and beta-cell function and insulin resistance.
- Liver blood samples (ALT(U/L), AST(U/L), FIB-4, INR, and hepatitis B viral load)
- Blood samples (CRP (mg/L), HbA1c (mmol/mL) and lipids (total cholesterol (mmol/L), total triglyceride (mmol/L), low density lipoprotein (LDL) (mmol/L), high-density lipoprotein (HDL) (mmol/L))
- Nail fold capillary assessment of vascularity (findings of the following: CD: Capillary Density-Distribution, CE: Capillary Enlargement, CT: Capillary Tortuosity, BC: Branching of the Capillaries, MH: Microhemorrhage, AA: Avascular Area, SH: Splinter Hemorrhage)
- Fibroscan measurements of hepatic steatosis and liver fibrosis (CAP (dB/m), fibrosis = TE-score (kPa))
- IPAQ-SF (participants self-assessed scoring of physical activity the previous 7 days resulting in a calculation of Metabolic Equivalence of Task (MET)-minutes)
- IQOLA SF-36: Quality of life questionnaire at baseline and post the intervention (participants self-assessed scoring of eight health concepts ranging from 0 to 100: physical functioning, role limitations due to physical health problems, role limitations due to personal or emotional problems, energy/fatigue, emotional well-being, social functioning, pain, and general health perception) at baseline to post-intervention
- 24-h recall food registration assessed by changes in amount and change of food divided into food categories and amounts of each category
- Liver biopsies change in the description of the degree of steatosis and liver inflammation

### Participant timeline {13}


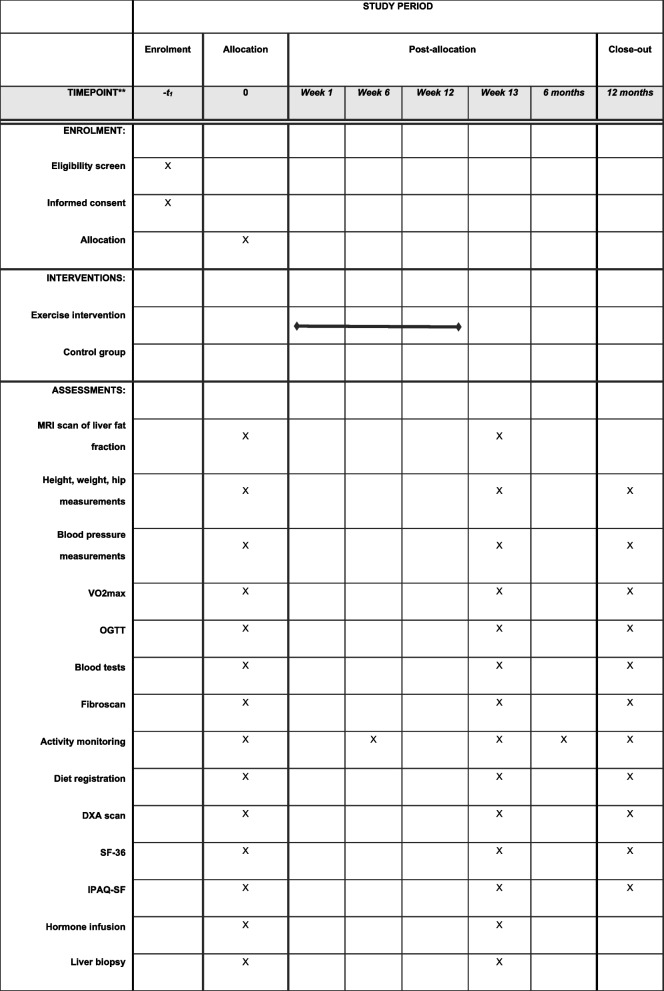


### Sample size {14}

The sample size for the primary outcome was calculated in relation to the fat fraction of the liver. To our knowledge, no-exercise intervention studies have been performed in patients with CHB. We used exercise training intervention studies in non-alcoholic-fatty liver disease (NAFLD) or sedentary, dysglycemic patients with measurements of MRI measurements of intrahepatic lipid content as the fat fraction to calculate the sample size. The one-sided test is chosen due to the current literature, which states that exercise training intervention generally reduces the fat fraction of the liver and does not increase the fat fraction of the liver [36, 61–63]. Assuming a SD of − 2,6 [36] and no change in the control group. An 80% chance of detecting a − 2.8% between-group change in liver fat fraction with a one-sided 0.05 significance requires *n* = 14 in each group.

### Recruitment {15}

The participants will be recruited through the outpatient clinic at the Department of Infectious Diseases, Copenhagen University Hospital, Hvidovre, at the Department of Infectious Diseases, Copenhagen University Hospital, Rigshospitalet, at the Department of Gastroenterology, Copenhagen University Hospital, Herlev, and through recruitment postings. Eligible participants will be identified by the primary investigator, who will contact the clinical staff treating the patient, who will request if the participants would like to receive information about the trial from the primary investigator. The recruitment postings will be posted in newspapers, through patient associations, on social media, by posters, and by flyers in local areas and hospital departments. Interested persons who have seen the recruitment postings can then contact the primary investigators by either email or phone. When including participants, the participants will receive a written and oral presentation of the trial in a closed environment without disturbances and the option for asking in-depth questions. The information will be given by the principal investigator. The trial participants will be given time—up to 2 weeks—to consider if they are willing to participate. If requested, an assessor can participate and ask questions to the primary investigator, including the trial participants. Inclusion will require written consent from trial participants before enrolment in the trial.

The flow chart of the trial will follow the CONSORT guidelines and will include the number of participants who (a) received written material and oral information about the trial, (b) were assessed for eligibility at prescreening, (c) were included in the trial, (d) attended CAP screening, (e) were randomized, and (f) were allocated to the two groups (intervention/control).

## Assignment of interventions: allocation

### Sequence generation {16a}

Randomization 1:1 is done using https://www.sealedenvelope.com/simple-randomiser/v1/lists with varying block randomization. No stratification is used.

### Concealment mechanism {16b}

Sealed envelopes with randomization intervention (1) or no intervention (0) will be made beforehand for the included trial participants by an outsider and investigators will not have knowledge of the content of the envelopes. The envelopes are sequentially numbered, opaque, sealed envelopes. The envelopes are opened sequentially, and each envelope will be irreversibly assigned to the participant after their first visit. Patients are given ID numbers accordingly to the time they have their first planned trial visit, e.g., FL01, FL02.

### Implementation {16c}

The randomization will be done according to the sealedenvelope.com algorithm, the participants will be enrolled after an outpatient visit, and on the first possible following day, the participants will show up for the informed consent. The primary investigator will open the sealed envelope at the end of the baseline examinations.

## Assignment of interventions: blinding

### Who will be blinded {17a}

We will strive to achieve blinded assessment of primary outcome and data analysis, but currently, due to limited funds, the primary investigator might be the one to assess the outcomes and make the data analysis and are unfortunately unblinded. If we do not succeed with the blinded assessment of outcome and data analysis, it will be described transparently in the trial report.

### Procedure for unblinding if needed {17b}

Not applicable, since there is no blinding of investigators during the trial and the blinding is intended only for the assessment of primary outcome and data analysis.

## Data collection and management

### Plans for assessment and collection of outcomes {18a}

Most outcomes are collected at baseline, at 13 weeks follow-up, and 1 year after the intervention. Apart from these additional outcomes for physical activity, measurements assessed by accelerometers are collected at baseline, at 6 weeks, at 13 weeks, 6 months after the intervention, and 1 year after the intervention. Data collecting regarding the exercise training sessions are collected at each session and kept in a file where it will be uploaded each week.

All data will be saved in an Easytrial database where data is validated by having 2 individual persons enter data (double entry) and the database will check for inconsistency.

### Plans to promote participant retention and complete follow-up {18b}

Participants are encouraged to continue the exercise training after the intervention. How to implement exercise training as part of their daily life is discussed at all participants’ 13 weeks’ follow-up. Participants are contacted at a 6-month follow-up after the intervention with a visit to receive the accelerometers. Participants are again contacted at 12 months for the last follow-up visit. There is no plan for promoting participant retention.

### Data management {19}

All patient-related information obtained during the trial will be handled by the Danish Data Protection Regulation and the Danish Data Protection Law. The blood samples will be registered from the hospital blood sample portal (Labka), and paraclinical observations will be obtained through EPIC (Epic Systems Corporation). No personal information will be transferred abroad. Collaboration with other Danish Universities will follow the Danish Data Protection Regulation and the Danish Data Protection Law. Personally, identifiable data will be stored in a password-secured web-based clinical trial management system EasyTrial database, which the Danish Data Protection Board has approved under the capital region. Blood samples and MRI scan results will be occurring in the patient files through the EPIC (Epic Systems Corporation).

### Confidentiality {27}

All signed consent forms will be kept in a locked cabinet at the CFAS behind 2 locked doors under the supervision of the primary investigator. Participant data on paper obtained in the trial is kept under the participants’ ID numbers. The de-identified data will be used in the data management and only authorized investigators will be able to access the paper documents. The de-identified data will be kept in a tracked restricted access network drive regulated by the Danish Data Protection Regulation. Publications from this trial will only contain de-identified information about trial participants. Data will not be freely available to other researchers but can be requested in a de-identified dataset to other researchers if found appropriate by the primary investigator.

### Plans for collection, laboratory evaluation, and storage of biological specimens for genetic or molecular analysis in this trial/future use {33}

#### How much and what

Blood will be drawn from the trial participants 5 times. Before the aerobic exercise training intervention, after 13 weeks and at 12 months follow-up, blood drawn before the intervention will be maximum of 300 mL over 2 days. Blood drawn after the intervention will be a maximum of 300 mL over 2 days. At 12 months follow-up, a maximum of 150 mL blood will be drawn. Liver biopsies will be collected before and after the intervention. At each collection, two samples of a maximum of 34.5 mg will be taken. This will include a total of a maximum of 138 mg liver tissue.

#### For what purpose

Some of the blood will be analyzed immediately for general tests like fasting glucose, fasting insulin, lipid status, HbA1c, hemoglobin, leucocytes and differential count, thrombocytes, CRP, ALT, basic phosphatase, LDH, bilirubin, INR, creatinine, carbamide, Na + , K + , creatine kinase, TSH, HBV serology, HBV DNA, HIV, and HCV. Other samples will be saved for analysis after the final participant has finished the trial period. The blood kept for further analysis will be kept in a research biobank at − 80 °C at CFAS. These blood samples will be analyzed for cytokines and hepatokines. Liver biopsies will be used for the histological description of the liver tissue. Any leftover tissue will be kept in a research biobank at the Department of Infectious Diseases, at Copenhagen University Hospital, Hvidovre under regulation by the Danish Data Protection Regulation and the Danish Data Protection Law.

#### Biobank for future research

When all tests are finalized, any excess blood or plasma and liver tissue will be stored in the research biobank at − 80 °C until the trial is finished (October 1st. 2023). After the trial period, the excess samples of blood or plasma, or liver tissue will be moved to a research biobank for future research, where they will be anonymized and stored for a further 10 years after the end of the trial (July 1st, 2033). The biobank for future research will be under the regulation by the Danish Data Protection Regulation and the Danish Data Protection Law. Any future studies that would like to use the biological material will need approval by the Danish Data Protection Regulation and the Danish Data Protection Law.

Trial participants can decline to donate blood for the biobank for future unspecified research without any consequences. Participants can at any time request to have their biological material destroyed. Such a request will lead to immediate destruction.

#### Responsibility and access

Principal investigator will have full access to the material in this period and be responsible for destroying all material after July 1st, 2033. The biological material will not leave Denmark. All regulations by the Danish Data Protection Regulation and the Danish Data Protection Law are kept.

## Statistical methods

### Statistical methods for primary and secondary outcomes {20a}

#### Analysis methods

The primary outcome (fat fraction) will be analyzed using a per protocol analysis according to the predefined compliance criteria. In addition, an intention to treat analysis will be carried out for all participants including those with low compliance. For both analyses, to detect if there is a significant change in liver fat fraction between the intervention and control group, analysis of covariance (ANCOVA) will be used including age, sex, and BMI as covariates. The estimated difference in mean change from baseline to end of the trial between the intervention and control group will be reported together with 95% confidence intervals and *p*-values.

Continuous secondary outcomes will be analyzed using ANCOVA with the same adjustments as for the primary outcome. Estimated differences in mean from baseline to end of the trial between the intervention and control group will be reported together with 95% confidence intervals and *p*-values. Model fit will be evaluated using graphical methods such as residual and QQ plots and, if necessary, outcomes will be log-transformed. For log-transformed outcomes, results will be back-transformed accordingly.

Statistical analyses will be carried out using R (R Core Team, 2021). R: A language and environment for statistical computing. R Foundation for Statistical Computing, Vienna, Austria. URL https://www.R-project.org/.

### Interim analyses {21b}

No planned interim analysis.

### Methods for additional analyses (e.g., subgroup analyses) {20b}

No planned subgroup analysis.

### Methods in analysis to handle protocol non-adherence and any statistical methods to handle missing data {20c}

The primary outcome will be assessed using a per protocol analysis including all participants who complete the compliance criteria defined by as follows: Conducting ≥ 70% of the 36 planned exercise training sessions with ≥ 50% of the assigned heart rate max (HR_max_) assessed by the supervising trainer in the exercise registration. For exercise training sessions where heart rate data is missing (e.g., due to a forgotten watch for supervised exercise training), the exercise training duration will be noted and the participant’s average intensity and time at different intensity zones will be imputed.

Due to the small sample size, missing data will not be imputed.

#### Missing data

The number/frequency of missing values for the primary and secondary outcome in each group at baseline and after the intervention will be provided.

### Plans to give access to the full protocol, participant-level data, and statistical code {31c}

This is an investigator-initiated trial. Access to the full protocol and participant-level data will be considered upon submission of a reasonable request and consent of the principal investigator.

## Oversight and monitoring

### Composition of the coordinating center and trial steering committee {5d}

This trial is including participants from 3 different departments and has two exercise training sites. The steering committee consists of the principal investigator together with professor, MD, Ph.D., Nina Weis, Clinical Associate professor, MD, Ph.D. Rikke Krogh-Madsen, and Professor, Dr. Med. Sci., Bente Klarlund Pedersen. Weekly or monthly meetings will be initiated by the principal investigator to discuss the progress of the trial.

### Composition of the data monitoring committee, its role and reporting structure {21a}

Due to the low-risk intervention with supervised HIIT exercise [50, 64] in a low-risk study population (no severe liver disease indicated by no cirrhosis/liver cancer or antiviral treatment, no coronary heart disease, no pregnancy), a data monitoring committee was not formed.

### Adverse event reporting and harms {22}

Adverse events and any serious adverse events are collected during the trial; any incidents are reported back to the principal investigator immediately and noted in a separate adverse events document. The primary investigator is responsible for handling the adverse event and referring the patients to relevant departments/clinicians/insurance if needed.

Any serious incidents and adverse events (SAE) will be reported immediately to the Danish Ethical Committee. Once a year during the entire trial period, the committee system must have sent a list of all SAE and serious incidents that occurred during the period. The list must be accompanied by 1–2 pages with a summary and assessment of the subjects’ safety. All patients will be covered by The Danish Patient Insurance Association for any injury that may occur as a direct consequence of trial-related procedures.

### Frequency and plans for auditing trial conduct {23}

An inspection or audit may take place, performed by the regulatory authorities. Inspectors will check the documents, logistics, records, and any other resources that the authorities consider to be associated with the clinical trial and that may be located at the trial site itself. But there is no planned external audit.

### Plans for communicating important protocol amendments to relevant parties (e.g., trial participants, ethical committees) {25}

The Danish Capital Regions committee on health research ethics will approve any protocol amendments and if required by the committee the participants will give their consent again for specific project changes. Any protocol amendments will be reviewed by the steering committee and participants will be informed if considered relevant. ClinicalTrials.gov will be updated with protocol amendments.

#### Deviations from registration on ClinicalTrials.gov

The following secondary outcomes is not included in the original outcomes in the ClinicalTrials.gov registration, as it was considered more of more explorative character: Nail fold capillary assessment of vascularity, fibroscan measurements of hepatic steatosis and liver fibrosis, IPAQ-SF, IQOLA SF-36, 24-h recall food registration, and liver biopsies change in the description of the degree of steatosis and liver inflammation.

### Dissemination plans {31a}

The trial results are planned to be published in international scientific journals with a focus on infectious diseases, aerobic exercise training, and hepatology and presented at national and international conferences.

## Discussion

This trial investigating high-intensity interval training in persons with chronic hepatitis B and hepatic steatosis is the first exercise training intervention trial performed on this group of patients. If aerobic exercise training reduces hepatic steatosis in this group of patients there might be an indication to encourage or offer aerobic exercise training systematically in the clinic. A recent meta-analysis concluded that persons with CHB have a higher prevalence of hepatic steatosis compared to controls [13]. Some suggest that hepatic steatosis when having CHB could be a potential benefactor in the suppression of viral replication and that the inflammation from viral replication potentially decreases the risk of developing hepatic steatosis [65]; however, this remains controversial. Having CHB and hepatic steatosis increases the risk of developing liver-related outcomes and all-cause mortality [66]. Current treatment guidelines from the European Study of the Liver (EASL) do include an initial screening for hepatic steatosis and metabolic disease but do not encourage any later screening for hepatic steatosis, or suggest treatment or medical care for persons with CHB and hepatic steatosis [67]. Living with CHB might at some point require treatment with antiviral medicine due to increases in viral load, fibrosis, or elevated liver enzymes. Antiviral treatment of CHB might have an effect on body fat mass, waist-to-hip ratio, and visceral fat mass, as suggested by Yao J. et al. [68], and are therefore exclusion criteria of this trial. With this trial, we hope to explore the effects of high-intensity interval training and investigate if there is a clinical indication for improvement of hepatic steatosis to indicate any potential for further studies and the use of aerobic exercise training as treatment in persons with chronic hepatitis B and hepatic steatosis.

## Trial status

Recruitment started in March 2022 using protocol version 1.3. We are currently recruiting. As of January 2023, 13 patients have been recruited. Recruitment is planned to end by June 30th, 2023.

## Data Availability

De-identified participant data can be made available, upon request, after the publication of the trial results. Further inquiries can be directed to the corresponding author.

## Abbreviations

AE: Adverse effect
ALT: Alanine aminotransferase
ANGPTL4: Angiopoeitin like protein 4
AST: Aspartate aminotransferase
BMI: Body mass index
CHB: Chronic hepatitis B
CVD: Cardiovascular disease
CAP: Controlled attenuated parameter
CRP: C-reactive protein
DNA: Deoxyribonucleic acid
DXA: Dual-energy X-ray absorption
EASL: European Study of the Liver
FGF-21: Fibroblast growth factor 21
GDF-15: Growth differentiation factor 15
HbA1c: Hemoglobin A1C
HBsAg: Hepatitis B surface antigen
HBV: Hepatitis B virus
HCV: Hepatitis C virus
HDV: Hepatitis D virus
HCC: Hepatocellular carcinoma
HIV: Human immunodeficiency virus
HRmax: Maximal heart rate
IDEAL-IQ: Iterative Decomposition of water and fat with Echo Asymmetry and Least squares estimation
IL-1β: Interleukin 1β
IL-4: Interleukin 4
IL-6: Interleukin 6
IL-8: Interleukin 8
IL-10: Interleukin 10
INR: International normalized ratio
IFN-γ: Interferon gamma
IPAQ-SF: International Physical Activity Questionnaire Short Form
LDH: Lactate dehydrogenase
MRI: Magnetic resonance imaging
NAFLD: Non-alcoholic fatty liver disease
OGTT: Oral glucose tolerance test
SAE: Serious adverse effect
SF 36: 36-Item Short Form Health Survey
TNF-α: Tumor necrosis factor α
TSH: Thyroid-stimulating hormone
T2DM: Type 2 diabetes mellitus
VO2_max_: Maximal oxygen consumption
